# Contamination and Spatial Variation of Heavy Metals in the Soil-Rice System in Nanxun County, Southeastern China

**DOI:** 10.3390/ijerph120201577

**Published:** 2015-01-28

**Authors:** Keli Zhao, Weijun Fu, Zhengqian Ye, Chaosheng Zhang

**Affiliations:** 1 School of Environmental and Resource Sciences, Zhejiang Agriculture and Forestry University, Lin’an 311300, China; E-Mail: kelizhao@zafu.edu.cn; 2 Geographic Information System Centre, Ryan Institute and School of Geography and Archaeology, National University of Ireland, Galway, Ireland; E-Mail: chaosheng.zhang@nuigalway.ie

**Keywords:** heavy metals, paddy soil, rice (*Oryza sativa* L.), spatial variation, spatial correlation

## Abstract

There is an increasing concern about heavy metal contamination in farmland in China and worldwide. In order to reveal the spatial features of heavy metals in the soil-rice system, soil and rice samples were collected from Nanxun, Southeastern China. Compared with the guideline values, elevated concentrations of heavy metals in soils were observed, while heavy metals in rice still remained at a safe level. Heavy metals in soils and rice had moderate to strong spatial dependence (nugget/sill ratios: 13.2% to 49.9%). The spatial distribution of copper (Cu), nickel (Ni), lead (Pb) and zinc (Zn) in soils illustrated that their high concentrations were located in the southeast part. The high concentrations of cadmium (Cd) in soils were observed in the northeast part. The accumulation of all the studied metals is related to the long-term application of agrochemicals and industrial activities. Heavy metals in rice showed different spatial distribution patterns. Cross-correlograms were produced to quantitatively determine the spatial correlation between soil properties and heavy metals composition in rice. The pH and soil organic matter had significant spatial correlations with the concentration of heavy metals in rice. Most of the selected variables had clear spatial correlation ranges for heavy metals in rice, which could be further applied to divide agricultural management zones.

## 1. Introduction

With the rapid development of industry and increasing release of agrochemicals into the environment, the potential accumulation of heavy metals in agricultural soils has caused a growing public concern about food security worldwide [1]. Heavy metals can pose long-term environmental and health implications because of their non-biodegradability and persistence [2,3,4]. Recent rapid economic growth in China has led to an increasingly serious problem of heavy metal contamination in agricultural soils [5,6].

Rice (*Oryza sativa* L.) is one of the most important agricultural crops in China. The quality of rice greatly affects human health, as consuming rice contaminated by cadmium (Cd), lead (Pb) and other metals can seriously deplete body stores of iron (Fe), vitamin C and other essential nutrients, leading to decreased immunological defenses, impaired psycho-social faculties and disabilities associated with malnutrition [7]. Therefore, it is of great importance to protect agricultural soils and ensure its sustainability.

A lot of work has been carried out on studying heavy metal bioavailability such as cadmium (Cd), copper (Cu), lead (Pb), chromium (Cr), zinc (Zn), and others in paddy soils [8,9,10,11]. Most previous studies on accumulation of heavy metals were focused on special field areas (e.g., industrial and mining regions). Little information is available on heavy metal accumulation in paddy fields at regional scales, and the spatial correlation between heavy metals in soil-rice system has seldom been investigated.

The Hangzhou-Jiaxing-Huzhou (HJH) Plain is one of the main rice production areas in China. The accumulation of heavy metals in soil of the study area could either directly endanger the natural soil functions, or indirectly endanger the biosphere by bioaccumulation in the food chain, and ultimately endanger human health [12]. In the past several decades, agrochemicals (phosphorus fertilizers) and organic fertilizers (pig manure and poultry litter) have been extensively applied in this region. The use of chemical fertilizers in HJH Plain increased from 1.25 Mg·ha^−1^ in 1975 to 3.23 Mg·ha^−1^ in 2000, and the use of pesticides increased from 82 to 175 Kg·ha^−1^, over the same period [12]. The repeated applications of these agrochemicals potentially contributed to the accumulation of heavy metals in agricultural soils as some of these fertilizers and pesticides contain heavy metals such as Cd, Pb, Zn [6]. HJH Plain is an important part of the Yangtze River delta, which is one of the most rapidly developing regions in China. Since the end of the last century, this region has experienced a rapid transition from a traditionally agricultural-based economy to an industrial economy, involving the establishment of engineering, electronics and other industries, most of which produce wastes and pollutants. Therefore, it is important to understand heavy metal pollution in HJH Plain and provide scientific knowledge for agricultural policy-makers.

The main objectives of this study were: (1) to characterize heavy metal concentrations and spatial patterns of surface soil and rice grains; (2) to study the spatial dependence and relationship of heavy metals in the soil-rice system; (3) to determine the soil properties influencing the availability of heavy metals to rice.

## 2. Experimental Section

### 2.1. Study Area and Sampling Site Description

**Figure 1:**
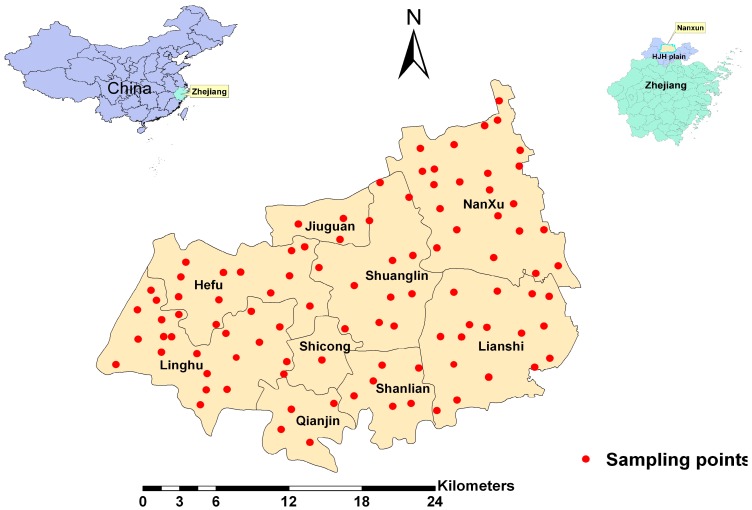
Location of the study area and sample sites.

This study was carried out in a main rice production area—Nanxun County—located in the center of HJH Plain, in north Zhejiang province, China (Figure 1). Nanxun covers an area of 716 km^2^ (120°40′ E to 120°29′ E, 30°38′ N to 30°56′ N), and has a population of 0.54 million. The soil type in the study area is a typical paddy soil for aquatic rice production. The term “paddy soil” in this paper is related to land use, not to any strict definition of soil type in pedology [13]. Nanxun has a subtropical marine monsoon climate with an average annual rainfall of 1230 mm and mean annual temperature of 15.7 °C.

A total of 100 pairs of rice grain and soil samples were collected from Nanxun County to provide a good spatial coverage of samples, based on a land use map at 1:50,000 scales. Each sample was the composite of at least 5 sub-samples within a distance of 10 m surrounding a specific sampling location. The rice and their corresponding soil samples (at 0–15 cm in depth) were collected from each site. At least 5 kg soil for each soil sample and 2.5 kg rice for each rice sample were collected. The longitudes and latitudes of the sampling points were recorded using a portable global position system (GPS). The distribution of sample locations in the study area is shown in Figure 1.

### 2.2. Laboratory Analyses

Soil samples were air-dried in the laboratory for several days at an ambient temperature. They were sieved to pass through a 2-mm nylon mesh for soil chemical and physical analyses. A portion of the samples were ground in an agate mortar to pass through a 0.149-mm (100 meshes) and stored in closed polyethylene bags for heavy metal concentrations and soil organic matter (SOM) analyses.

Soil properties were determined according to the standard methods [14]. Soil pH and electrical conductivity (EC) were analyzed in an aqueous suspension (1:2.5 and 1:5 soil-water ratio, respectively). Soil organic matter (SOM) was determined using the potassium dichromate wet combustion procedure. Soil particle size distribution (sand, silt and clay content) was analyzed using the hydrometer method.

Total heavy metal concentrations in soils were determined following digestion using strong acids of HF, HNO_3_ and HClO_4._ Soil Cd concentration was measured using graphite furnace atomic absorption spectroscopy (GFAAS, PerkinElmer AA800, Waltham, MA USA) with NH_4_H_2_PO_4_ and Mg(NO_3_)_2_ as its matrix modifier, and the detection limit was 0.002 mg·L^−1^. Copper, Ni, Pb and Zn concentrations were determined by flame-atomic absorption spectroscopy (FAAS, PerkinElmer AA800), and the detection limits for Cu, Ni, Pb, and Zn were 0.05, 0.1, 0.2, and 0.05 mg·L^−1^, respectively.

Rice grain samples were oven-dried at 105 °C for 1 h, then at 70 °C to constant weight. Hull was removed from rice. Then the white rice samples were comminuted using a pulverizer, ground to pass through a 0.149-mm (100 meshes) sieve and stored in closed polyethylene bags for further heavy metal concentration analysis. Rice grain samples were digested using HNO_3_ and H_2_O_2._ Cd, Cu, Ni and Pb concentrations were determined by GFAAS (Perkin Elmer AA800), and the detection limits for the corresponding metals were 0.002, 0.014, 0.07, and 0.05 mg·L^−1^. Zn concentrations were determined by FAAS (Perkin Elmer AA800) and the detection limit was 0.05 mg·L^−1^. In this study, the concentrations of Pb in the extracted solution were below the limit of detection (0.05 mg·L^−1^). Therefore, the Pb content in rice was not detected.

The accuracy of determinations was verified using the Chinese standardized reference materials (GSS-4 and GSS-15 for soil samples; GBW (E) 080684 for rice samples). All samples were measured in duplicate.

### 2.3. Evaluation Method for Soil Pollution and Standard

To evaluate heavy metal pollution in soils, single factor pollution index (SFPI) was first applied [15]. It was calculated using the following formula:

(1) $$P_{i}=C_{i}/S_{i}$$

where *P_i_* is pollution index of pollutant *i*, *C_i_* is the measured value of *i*, *S_i_* is the guideline value of *i*. When *P_i_* is less than 1, it stands for no heavy metal pollution, when *P_i_* is larger than 1, it represents heavy metal pollution in the studied area.

While SFPI quantifies the individual heavy metal pollution in soils, the Nemerow multi-factor pollution index measures the overall heavy metal pollution. Nemerow multi-factor pollution can be expressed as:

(2) $$I=\sqrt{\left({P_{i}^{2}}_{Max}+{P_{i}^{2}}_{Ave}\right)/2}$$

where *I* is Nemerow multi-factor pollution index at location *i*, *P_i Max_* and *P_i Ave_* represent the maximum and average values of SFPI, respectively. Based on Nemerow multi-factor pollution index, the environmental quality is divided into five levels, including Clean level (*I* ≤ 0.7), Precaution level (0.7 < *I* ≤ 1.0), Light pollution level (1.0< *I* ≤ 2.0), Moderate level (2.0 < *I* ≤ 3.0), Heavy Pollution level (*I* > 3.0) [15].

The background values of heavy metals from agricultural soils in Zhejiang Province [16] and Environmental Quality Standards for Soils (EQSS) in China [17] (Table 1) were used as guideline values for the assessment of heavy metal accumulation and pollution, respectively, in the study area. The EQSS includes three levels, and the second one was adopted, as the guideline values for agricultural production.

**Table 1:** The background values of heavy metals in soils in Zhejiang and environmental quality standard for soils in China, heavy metal standards for food safety (mg·kg^−1^).

Heavy Metals	Background Values in Zhejiang	The First Level	The Second Level			The Third Level	Food Safety Standards (in Rice)
			pH < 6.5	pH 6.5–7.5	pH > 7.5	pH > 6.5	
Cd	0.129	0.2	0.3	0.3	0.6	1.0	0.2
Cu	30.54	35	50	100	100	400	10
Ni	36.48	40	40	50	60	200	10
Pb	30.46	35	250	300	350	500	0.2
Zn	107.79	100	200	250	300	500	50

### 2.4. Geostatistical Analysis

A variogram (or semi-variogram) is used to measure the spatial variability of a regionalized variable and provide the input parameters for the spatial interpolation of kriging. Detailed information of geostatistics is widely available in textbooks [18,19]. In this study, disjunctive kriging was applied. The disjunctive kriging is the principal technique to estimate the probability that the true values of soil heavy metals at an unsampled location exceed a specified threshold. It is based on the assumption that the data are a realization of a process with a second order stationary bivariate distribution. The assumption of second order stationarity means that the covariance function exists and that the variogram is therefore bounded. It is assumed that the concentration of a heavy metal is a realization of a random variable *Z*(*x*), where *x* denotes the spatial coordinates in two dimensions. If a threshold concentration *z_c_* is defined, marking the limit of what is acceptable, then the scale is dissected into two classes which is less and more than *z_c_*, respectively. The value 0 and 1 can be assigned to two classes. A new binary variable, or indicator, which is denoted by Ω [*Z*(*x*) ≥ *z_c_* ]. At the sampling points the values of Z are known, and so the values 0 and 1 can be assigned with certainty. Elsewhere, one can at best estimate Ω [*Z*(*x*) ≥ *z_c_* ]. In fact, it is necessary to do this in such a way that the estimate at any place *x*_0_ approximates the conditional probability, given the data, that *Z*(*x*) equals or exceeds *z_c_* [19].

The cross-correlogram was applied to determine spatial correlations between two variables separated by a distance *h*. At zero distance, the cross-correlogram ρ_12_ (0) is equal to the Pearson correlation coefficient [18,20]. The cross-correlogram can be used to describe the similarity of spatial patterns, which is similar with ρ_12_ (0) equal to 1 and oppositely similar with when ρ_12_ (0) equals to −1 [21]. A high cross-correlogram value indicates a strong correlation. It is calculated as follows [20]:

(3) $$\rho_{12}(h)=\frac{1/n\sum_{i=1}^{n}Z_{1}(x_{i})Z_{2}(x_{i}+h)-m_{1-h}m_{2+h}}{\sqrt{\sigma_{1-h}^{2}\sigma_{2+h}^{2}}}$$

where *Z*_1_(*x_i_*) is the value of variable 1 at location x_i_; *Z*_2_(*x_i_ + h*) is the value of variable 2 at a location separated by distance *h* from location *x_i_*; *m*_1 *− h*_ and *m*_2 *+ h*_ are the means of variable 1 and variable 2, respectively; σ^2^_1 *− h*_ and σ^2^_2 *+ h*_ are the variances of variable 1 and variable 2, respectively; and *n* is the number of data pairs used tocalculate the cross-correlogram at each distance *h*.

Three parameters (*r*, range, and shape) in the cross-correlogram are important to describe the spatial correlation between two variables. The cross-correlogram value at zero distance indicates the strength of the relationship. The spatial correlation range gives the distance over which two variables are correlated. The shape of the cross-correlogram indicates how fast the correlation between two variables diminishes with distance.

### 2.5. Data Analysis with Computer Software

In linear geostatistical methods, a normal distribution for the studied variable is desired to obtain more reliable results [19]. In this study, a statistical test of the Kolmogorov-Smirnov (K-S) method together with skewness and kurtosis values were applied to evaluate the normality of data sets. The logarithmic transformation was performed on raw data sets which did not follow a normal distribution.

A specific number of values were extracted from the interpolated distribution maps by a completely randomized sampling. The density was about 1 km^2^ per sample, so that 750 samples were collected for cross-correlogram analysis. The descriptive parameters were calculated using SPSS^®^ for Windows (version 18.0). The geostatistical analysis was carried out with GS＋ for Win. 7.0. All maps were produced using GIS software ArcMap^®^ (version 9.2).

## 3. Results 

### 3.1. Heavy Metal Concentrations in the Soil-Rice System

The representative descriptive statistics for heavy metal concentrations in soils and rice are listed in Table 2. The total concentrations of Cd in soils were very variable, ranging from 0.12 mg·kg^−1^ to 0.78 mg·kg^−1^, with an average of 0.21 mg·kg^−1^. Its kurtosis and skewness values were highly positive (Table 2), indicating the positively skewed distribution. Similar phenomena were observed for total concentrations of Cu and Zn in soils. Among the heavy metals, the total Zn in soils had the largest mean value (106.82 mg·kg^−1^). The coefficient of variation (CV) values, which were used for description of global variability (relative to local), for total Cd, Cu, Ni, Pb and Zn in soils, were 34.96%, 23.97%, 21.00%, 16.08% and 28.13%, respectively. All the heavy metals had moderate variability in soils.

**Table 2:** Descriptive statistics for heavy metals in soils and rice (mg·kg^−1^).

Metals	Mean	SD	Min	Max	Kurtosis	Skewness	CV (%)
Cd_soil_	0.21	0.07	0.12	0.78	33.90 (0.32)	4.66 (0.29)	34.96
Cu_soil_	31.06	7.45	18.80	82.31	22.22 (0.82)	3.50 (0.21)	23.97
Ni_soil_	32.14	6.75	18.49	55.59	0.38	0.19	21.00
Pb_soil_	33.20	5.34	20.19	51.71	1.07	0.65	16.08
Zn_soil_	106.82	30.05	61.55	266.93	9.30 (0.37)	2.33 (0.39)	28.13
Cd_rice_	0.011	0.015	0.003	0.103	28.13(1.67)	5.04 (1.07)	126.88
Cu_rice_	2.49	0.74	1.06	4.23	−0.13	0.22	29.62
Ni_rice_	0.125	0.173	0.025	1.403	36.43 (1.47)	5.66 (0.81)	138.64
Zn_rice_	14.28	2.70	9.45	21.97	−0.21	0.59	18.93

Cd_soil_, Cu_soil_, Ni_soil_, Pb_soil_, Zn_soil_, heavy metals in soils; Cd_rice_, Cu_rice_, Ni_rice_, Zn_rice_, heavy metals in rice. Kurtosis and skewness values in brackets were calculated after log-transformation. CV% was SD in percent to the mean.

The concentrations of Cd in rice ranged from 0.003 mg·kg^−1^ to 0.103 mg·kg^−1^, with an average of 0.011 mg·kg^−1^. Both Cd and Ni in rice had highly positive kurtosis and skewness values. Their log-transformed data passed the normality test (K-S*_p_* > 0.05, Table 2). Meanwhile, they had relatively high CV values (Table 2). The concentrations of Cu ranged from 1.06 mg·kg^−1^ to 4.23 mg·kg^−1^. The concentrations of Zn varied between 9.45 mg·kg^−1^ and 21.97 mg·kg^−1^. Compared to the corresponding variables in soils, the heavy metals in rice had larger CV values, except for Zn. This may be related to the influences of soil physical-chemical properties and rice genotypes on heavy metals in rice.

### 3.2. The Accumulation and Pollution of Heavy Metals in Paddy Fields

Compared with the heavy metal background values in Zhejiang Province, the average SFPI values of Cd, Cu, Pb in soils were higher than 1 (Table 3). The accumulation ratio followed the order of Cd > Pb > Cu > Zn > Ni. For total Cd in soils, about 99% of the soil samples exceeded the background value, indicating obvious Cd accumulation in paddy soils of the study area. Compared with second grade standardized value of EQSS in China, the average SFPI values were lower than 1, indicating that the overall soil quality in the study area was safe for agricultural production. But attention should be paid to the particular locations where the concentrations of heavy metals such as Cd and Ni, exceeded the guideline values (Table 3).

The Nemerow multi-factor pollution index results (Table 4) showed that 88% of the soil samples belonged to light pollution level based on background values, indicating the wide spread of heavy metal accumulation in study area. While 43% and 54% of soil samples belonged to clean and precaution levels, respectively, based on second grade standardized value of EQSS, indicating the potential heavy metal pollution in Nanxun. All the heavy metals in rice remain a safe level for human being consumption (Table 1 and Table 2).

**Table 3:** The evaluated results of single factor pollution index (SFPI) for heavy metals in soils.

Metals	Background Value as Critical Value				Second Grade Standardized Value as Critical Value			
	Mean	Min	Max	Ratio (%)	Mean	Min	Max	Ratio (%)
Cd	1.65	0.91	6.06	99.00	0.71	0.39	2.61	6.00
Cu	1.02	0.62	2.70	49.00	0.62	0.38	1.65	2.00
Ni	0.88	0.51	1.52	27.00	0.80	0.46	1.39	9.00
Pb	1.09	0.66	1.70	69.00	0.13	0.08	0.21	0
Zn	0.99	0.57	2.48	40.00	0.53	0.31	1.33	2.00

**Table 4:** The evaluated results of heavy metal pollution in soils (%).

Standard	Clean *I* ^*^ < 0.7	Precaution Level 0.7 < *I* < 1	Light Pollution 1 < *I* < 2	Moderate Pollution 2 < *I* < 3	Heavy Pollution *I* > 3
Background Value	0	5	88	6	1
Second grade Standardized Value	43	54	2	1	0

*I* ^*^ is Nemerow multi-factor pollution index.

### 3.3. Transfer Rate of Heavy Metals from Soil to Rice

**Figure 2:**
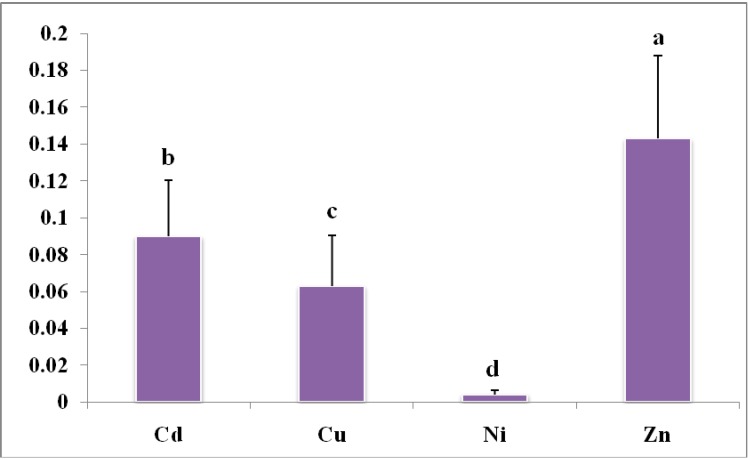
Enrichment index of heavy metal concentrations between soil and rice in paddy field. Different capital letters mean significantly different at the 0.05 level.

In order to understand the relationship of heavy metals in soil–rice system, the enrichment index (EI) was applied in this study, which was defined as the metal concentration in rice divided by that in soil [13]. Enrichment index provides a useful indication of the metal availability from soil to plants [22]. The result of EI for each heavy metal was shown in Figure 2. The average values of EI for Cd, Cu, Ni and Zn were 0.0829, 0.0567, 0.0041 and 0.1429, respectively, indicating the availability of heavy metals to rice was generally in the order of Zn >Cd >Cu >Ni. The EI varied significantly (*p* < 0.05) among heavy metals in this study.

### 3.4. Spatial Structure and Spatial Distribution

The semivariance models and their key parameters are given in Table 5. The best-fit theoretical model for the experimental semivariogram was chosen based on the highest decision coefficient value (*r^2^*) of all theoretical models. The Cd, Ni and Pb in soils were best fit with spherical models. The Cu and Zn in soils were best fit with exponential models (Table 5). The Cd, Cu, Ni and Zn in rice were all best fit with exponential models (Table 5).

The “nugget/sill” ratios for Cd, Cu, Ni, Pb and Zn in soils were 25.6%, 29.6%, 33.1%, 48.9% and 49.9%, respectively. All the heavy metals in soils had moderate spatial dependence. The “nugget/sill” ratios for Cd, Cu, Ni and Zn in rice were 33.4%, 42.6%, 13.2% and 14.5%, respectively (Table 5). Cd and Cu in rice had moderate dependence and Ni and Zn had strong spatial dependence. The ranges of heavy metals in soils were longer than 10 km, except for Cd. Compared to the Ni and Zn in rice, Cd and Cu in rice had longer ranges.

**Table 5:** The theoretical semivariogram models and their corresponding parameters for heavy metals in soils.

Metals	Distribution Type	Models	(Nugget) C_0_	(Sill) C_0_ + C	(Nugget/Sill %) C_0_/(C_0_ + C)	Range A (km)	*R^2^*
Cd_soil_	Log-normal	Spherical	0.011	0.043	25.6	0.80	0.986
Cu_soil_	Log-normal	Exponential	0.014	0.047	29.6	31.68	0.972
Ni_soil_	Normal	Spherical	18.50	55.82	33.1	21.07	0.960
Pb_soil_	Normal	Spherical	15.91	32.55	48.9	22.43	0.876
Zn_soil_	Log-normal	Exponential	0.030	0.059	49.9	13.26	0.796
Cd_rice_	Log-normal	Exponential	0.252	0.775	33.4	21.33	0.824
Cu_rice_	Normal	Exponential	0.386	0.907	42.6	12.70	0.892
Ni_rice_	Log-normal	Exponential	0.050	0.380	13.2	3.93	0.828
Zn_rice_	Normal	Exponential	1.06	7.31	14.5	4.59	0.913

Cd_soil_, Cu_soil_, Ni_soil_, Pb_soil_, Zn_soil_, heavy metals in soils; Cd_rice_’ Cu_rice_, Ni_rice_, Zn_rice_, heavy metals in rice.

**Figure 3:**
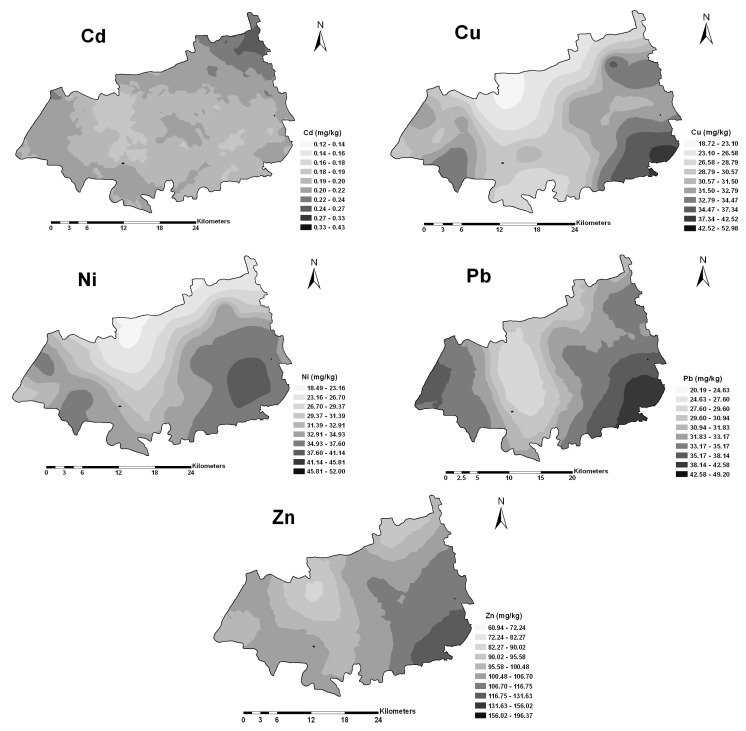
Spatial distribution maps of heavy metals in soils.

The Cu, Ni, Pb and Zn in soils had similar spatial distribution patterns, with high concentrations located in the south-east and south-west parts of the study area, and low concentrations in the center (Figure 3). Compared to other heavy metals, Cd in soils showed a different spatial pattern. Its high concentration values were observed in the northeast part of Nanxun, with some small scale patterns of medium and low concentrations, which were in line with its short range in the semivariogram model.

**Figure 4:**
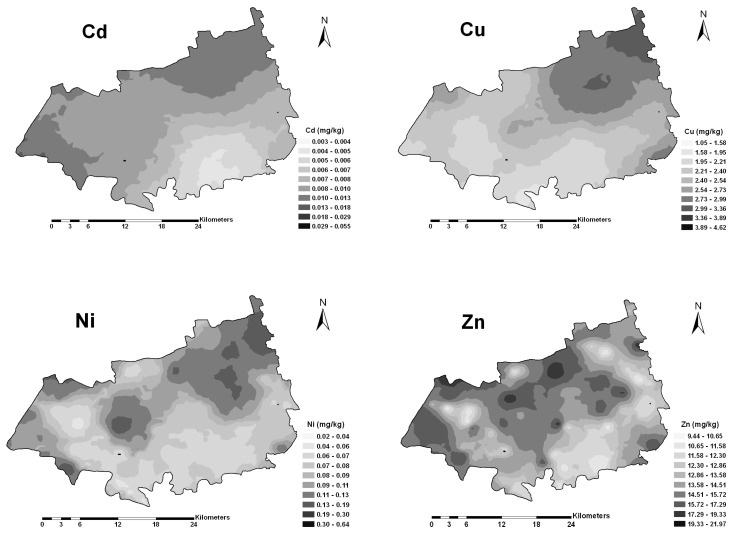
Spatial distribution maps of heavy metals in rice.

**Figure 5:**
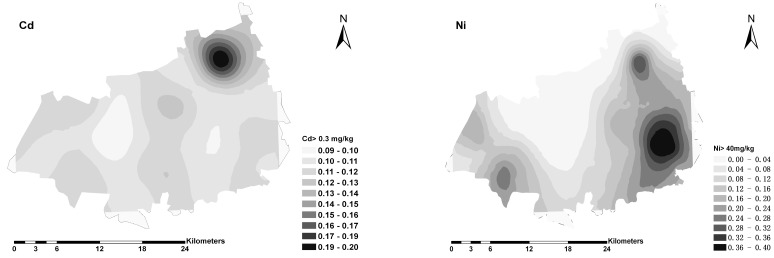
The estimated probability map of Cd and Ni.

The spatial distribution of Cd in rice showed a north-to-south trend with high concentrations located in the north and low concentrations in the south (Figure 4). Copper and Ni in rice shared similar spatial patterns with high values in the northeast and low values in the southwest part. Zinc in rice showed high concentrations in the northwest part. Meanwhile, Cd and Cu in rice showed large spatial patches while Ni and Zn in rice showed small spatial patches. Meanwhile, the estimated probability of excess for Cd, Cu, Ni and Zn, defined by the thresholds in Table 1, was kriged by disjunctive kriging and given in Figure 5 (Cd and Ni as examples).

For soil Cd, the map showed that the areas with high risk were mainly located in northeast part of Nanxun county, where the estimated probability Ω [Cd ≥ 0.3 mg/kg ] reached 0.19–0.2. The probability map of Ni exceeding the guide value 40 mg/kg exhibited many risk patches. Especially, the highest risk areas were distributed in southeastern part of the study area. Compared to Cd and Ni, the probability of excess for soil Cu and Zn were relevantly low (not shown).

### 3.5. Spatial Correlation between Heavy Metals in Rice and Soil Properties

**Figure 6:**
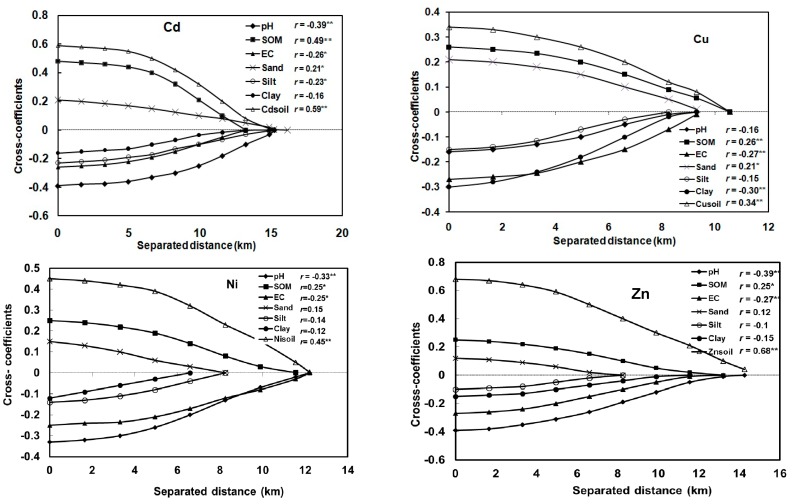
Cross-correlograms for each heavy metal between heavy metal in rice and soil properties, total heavy metal in soil. (^**^ Correlation is significant at the 0.01 level; ^*^ correlation is significant at the 0.05 level).

In order to understand the potential effects of total soil heavy metals and soil properties on the availability of heavy metals to rice, cross-correlograms were produced to quantitatively determine their spatial correlations (Figure 6).

The maximum cross-correlogram values were observed at 0 distance, which are equal to the Pearson’s correlation coefficients (*r* values). Heavy metals in rice were all positively spatially correlated with SOM, sand contents, corresponding heavy metals in soils, and negatively spatially correlated with soil pH, EC, silt and clay contents. In the cross-correlogram for Cd, compared to soil texture (sand, silt and clay), the pH, SOM and EC had stronger cross-correlations with the Cd in rice. The similar phenomena were observed in the cross-correlograms for Ni and Zn. All the heavy metals in rice had significant spatial correlation with their corresponding counterparts in soil, among which element Zn had the strongest cross-correlation. Meanwhile, most of the soil properties had a clear spatial correlation range with heavy metals in rice. As the distance increased, the cross-correlogram values gradually decreased to 0 (Figure 6). Compared to other soil properties, pH generally had the longest spatial correlation distance with heavy metals in rice.

## 4. Discussion

Compared to the background values of heavy metals in soils (Table 1), Cd, Cu, Pb and Zn (Table 3 and Table 4) were obviously enriched in the study area. In order to improve the yield of rice grain, a large amount of chemical fertilizers (increased from 1.25 T·ha^−1^ to 3.23 T·ha^−1^) has been applied for the past three decades. This amount of fertilizers exceeded the maximum guideline for safe agricultural production [23]. It was found that the concentrations of Cd, Cu, Pb and Zn in chemical fertilizers were 0.0005–0.5 mg·kg^−1^, 0.41–11.6 mg·kg^−1^, 0.0008–0.93 mg·kg^−1^ and 4.87–348.2 mg·kg^−1^, respectively [24]. In this study, all samples were collected from agricultural fields which received chemical fertilizers, while there was no fertilizer application in the area where background values of heavy metals in soils were determined. Meanwhile, farmers applied pesticides in order to control plant diseases and insects. For the single cropping of rice, repeated pesticide applications (8 times) were carried out during the growing seasons [25]. Plumlee [26] reported that excessive usage of pesticides and herbicides could lead to Cu, As, Pb and Zn accumulation in topsoils of agricultural fields. The long-term application of fertilizers and pesticides may be one of the main sources for heavy metal accumulation in soils in the study area [6,12].

In general, heavy metal concentrations in rice remained at a safe level in the study area (Table 2). The concentrations of Cd in rice ranged from 0.003 mg·kg^−1^ to 0.103 mg·kg^−1^, which were lower than the guideline value for food safety (Table 1). Heavy metal concentrations in rice were comparable with those in the areas under traditional agricultural management [13,27], but lower than those in the areas influenced by industrial activities such as an e-waste dismantling areas [28], or irrigated with sewage [29]. Attention should be paid to the locations where heavy metals in soils exceeded the second level of EQSS, as heavy metal concentrations in rice of these areas were also relatively high.

The availability of heavy metals to rice was significantly (*p* < 0.05) different among heavy metals. These results were in line with other studies. Kashem and Singh [30] reported that the accumulation of Cd and Zn was higher than that of Ni in rice. Heavy metal concentrations of Cd and Zn in rice increased faster with increasing metal concentrations in paddy soil than that of Cu and Pb [31]. The EI value of Zn was higher than that of Cd (Figure 2). This finding was different with a previous study [13]. This was probably related to the different rice types, as the rice genotypes were the main factors influencing the transfer and bioavailability of heavy metals in the soil-rice system [32,33]. The main rice type in Nanxun County was *Japonica* Rice, while the main rice type in Wenling was Hybrid Rice [13]. Zeng *et al.* [33] have reported that Hybrid Rice accumulated more heavy metals than *Japonica* Rice under the same soil conditions.

The parameters of semivariogram models provide information of the spatial variability, including intrinsic variability and extrinsic variability. The intrinsic variability is considered to be mainly from natural variation such as parent material, while extrinsic variability is mainly due to human activities [6,34,35]. As a rough guide, the variable is considered to have a strong spatial dependence if the “nugget/sill” ratio is < 25%, a moderate spatial dependence if this ratio is between 25% and 75%, and weak spatial dependence if the ratio is >75% [22]. Usually, strong spatial dependence of soil variables may be controlled by intrinsic factors, and weak spatial dependence may indicate that variability is controlled more by extrinsic factors [36,37]. Heavy metals in soils were moderately spatially dependent in this study (Table 5). This was attributed to both intrinsic factors such as soil texture and extrinsic factors such as agricultural activities, industrial sources and other anthropogenic activities. The results were in line with previous studies on the spatial structures of heavy metals in agriculture soils [34,35,38]. In geostatistical theory, the range of a semivariogram is the measure of spatial extension within which autocorrelation exists [39]. The range values of heavy metals in soil (except Cd) were longer than 10 km, which were much longer than the sampling interval. This suggested that the current sampling design is good enough to reveal spatial distribution features of heavy metals in soils.

The kriging estimates can be mapped to reveal the overall trend of the data [40]. Maps provide useful visual display of the spatial variability and can be used to represent and summarize soil properties [41]. As shown in Figure 3, the high concentrations for Cu, Ni, Pb and Zn were located in the southeast part of the study area, where the paddy fields were contaminated to some degree (Table 4). Based on our survey, it was clearly found that many industries are distributed in the study area, which included mechanical and electric production, leather and plastic production, dye, and others [42]. These industries are the likely sources for the extrinsic factors leading to these high concentrations of heavy metals in soils. There were a few small-scale patterns of high Cd concentrations in the north part of the study area, which was related to industrial point pollution sources such as coal-fired heating station and Ni-Cd battery production plants.

Compared with the spatial distribution patterns of heavy metals in soils, differences were observed for the corresponding heavy metals in rice (Figure 3 and Figure 4), indicating that other factors may also play an important role in the transfer of heavy metals from soils to rice. Cross-correlograms further quantified the spatial correlation between the heavy metals in rice and soil properties, and provided information for delineating the size of potential management zones. Figure 5 revealed that high SOM would increase the accumulation and availability of heavy metals in rice (*p* < 0.01 or *p* < 0.05). In contrast, high soil pH and EC contents decreased the accumulation and availability. Furthermore, pH and SOM showed higher spatial correlation with heavy metals in rice, except for Cu. Soil pH influenced the dissolution of heavy metals, particularly in acid paddy field [43]. Low pH may result in increased solubility and high availability of heavy metals for rice [13]. Previous studies investigated the effect of soil properties on metals uptake by plants [6,44]. Soil pH is a significant factor controlling uptake of heavy metals, and above all, is perhaps the most important factor [31,45]. The SOM was another soil property that influenced the distribution of heavy metals in soils and the bioavailability of heavy metals to rice. The effect of SOM on the availability of heavy metals could be due to lower solubility of heavy metals in soils, and the availability of heavy metals decreased with increasing SOM [30,33]. Soil EC also played a role in the availability of heavy metals in the soil-rice system, as negative correlations were found between EC and heavy metals. The observed cross-correlograms (Figure 5) indicated that soil properties did influence the transfer of heavy metals from the soils to rice, which was not limited to individual sites but encompassed to all paddy fields. The influence of soil properties on the availability of heavy metals in rice varied among different heavy metals. Compared to other heavy metals, Cu in rice was weakly correlated with soil pH and OM, implying that the transfer of Cu in paddy soils and rice may be affected by other factors, which needs further studies.

## 5. Conclusions

Based on the guideline values for heavy metal pollution, the paddy fields of Nanxun showed Cd, Cu, Ni and Zn contaminations, while the rice remained at a safe level. The long-term application of fertilizers and pesticides, and industrial activities were the main pollution sources in the study area. Soil properties, especially pH and SOM, played an important role in the availability of most heavy metals to rice plants in the paddy fields. Heavy metals in soils and rice had clear spatial patterns. Such information, combined with soil properties, could be used for rational site-specific management in paddy fields.
